# Evidence-Based Practice in Primary Care Occupational Therapy: A Cross-Sectional Survey in Sweden

**DOI:** 10.1155/2018/5376764

**Published:** 2018-10-28

**Authors:** Ann-Charlotte Lindström, Susanne Bernhardsson

**Affiliations:** ^1^Närhälsan Rehabilitation Sörhaga, Alingsås, Sweden; ^2^Närhälsan Research and Development Primary Health Care, Region Västra Götaland, Gothenburg, Sweden; ^3^Institute of Neuroscience and Physiology, Department of Health and Rehabilitation, Unit of Physiotherapy, University of Gothenburg, The Sahlgrenska Academy, Gothenburg, Sweden

## Abstract

**Introduction:**

Understanding of attitudes, knowledge, and behaviour related to evidence-based practice (EBP) and guidelines in Swedish occupational therapy is limited. The study aims were to investigate attitudes, knowledge, and behaviour related to evidence-based practice and guidelines of Swedish occupational therapists in primary care.

**Methods:**

A web-based survey of 94 Swedish primary care occupational therapists (response rate 53.7%). Data were analysed using logistic regressions.

**Results:**

Attitudes towards EBP and guidelines were highly positive (97%–98%). About half of the respondents reported confidence in finding and using evidence. Almost two-thirds reported being aware of guidelines and 47% knowing where to find guidelines. Four-fifths stated that they had easy access to guidelines and 75% that they used guidelines frequently. Men were more likely to feel confident to find research (OR 8.58, 95% CI 1.03 to 71.66; *p* = 0.047) and have easy access to guidelines (OR 9.10, 95% CI 1.94 to 42.83; *p* = 0.005). Occupational therapists older than 50 years were more likely to integrate patient preferences with guideline use (OR 6.44, 95% CI 1.14 to 36.57; *p* = 0.035). Few reported reading scientific articles, and many expressed uncertainty in finding research. The main barrier for using guidelines was reported to be lack of time.

**Conclusion:**

Although attitudes among primary care occupational therapists towards EBP are positive and a large proportion report using guidelines, many state that they want to learn more and improve their evidence-based practice skills. The findings suggest that education measures need to be taken to address the identified shortcomings.

## 1. Introduction

Evidence-based practice (EBP) means the integration of best available evidence from clinical research with clinical expertise and patients' preferences [1]. In recent years, EBP has become an expectation of most healthcare professionals and a vehicle to advance the profession and ensure that occupational therapists deliver quality services to their patients [2, 3]. Applying EBP ensures that clinicians use effective interventions to achieve desired outcomes and contributes to best quality care [4].

However, despite the spread of EBP during recent decades, research findings are not routinely used in occupational therapy practice [5–8]. To do so requires that clinicians have the necessary knowledge and skills to access, appraise, and apply research evidence in their clinical decision making, as well as sufficient time [9]. Applying EBP in occupational therapy is particularly challenging since research evidence for most interventions is limited [10]. Occupational therapy research has traditionally been more interpretive in approach, frequently involving case-based action with qualitative outcomes rather than effectiveness studies of interventions, and the profession has been slow to adopt research-informed practice [11]. Many factors affect whether occupational therapy practice is based on research evidence: individual factors such as attitude, preferences, educational level, context, knowledge, and skills, as well as organisational factors such as attitudes of managers and colleagues. Factors relating to both the individual and the organisation, e.g., support and time available to search for research evidence, are particularly important [10].

Knowledge and skills related to EBP vary among different countries, and various barriers for applying EBP have been identified [3–5, 9, 12]. A systematic review showed that attitudes toward EBP among occupational therapists in different countries vary [5]. While several of the included studies reported positive attitudes to various extents, some reported negative attitudes, particularly related to the perceived difficulty of understanding and applying EBP in clinical practice. In Sweden, hospital-employed occupational therapists have been shown to hold positive attitudes toward EBP, but lack of time is a key obstacle to integrating the research component of EBP into practice [13]. No study on EBP has been performed in a Swedish primary care occupational therapy context.

The use of clinical practice guidelines is a widely used strategy to implement EBP, and high quality guidelines provide evidence-based recommendations to support the clinician in choosing effective interventions. Over time, these types of guidelines have shifted from opinion-based to research-informed [14], thus constituting an excellent point of departure for integrating research findings with the other EBP components. An increasing number of guidelines are being produced that include recommendations for occupational therapy, for example, by the American Association of Occupational Therapists [15], the British Royal College of Occupational Therapists [16], and the Swedish National Association of Occupational Therapists [17]. However, implementation, uptake, and use of guidelines tend to vary based on different factors related to the guideline itself, the user/clinician, the patient, and the practice context [18].

There is a lack of knowledge about attitudes, access, knowledge, determinants, and use of EBP and clinical practice guidelines in primary care occupational therapy in Sweden, and whether any associations exist with demographic characteristics. Knowledge about these factors is important so that current trends in occupational therapy practice can be identified [12]. This knowledge also provides a basis for developing effective implementation strategies for clinical practice guidelines in order to increase the extent to which occupational therapy practice is evidence-based. Primary care occupational therapists in Sweden treat a wide variety of conditions and practice autonomously, without need for a doctor's referral, and applying EBP and using guidelines in everyday patient work are important so that patients are treated with effective methods and receive equal care. Therefore, the aim of this study was to investigate attitudes, knowledge, and behaviour related to EBP and guidelines among Swedish occupational therapists in primary care.

## 2. Methods

### 2.1. Participants and Setting

Eligible participants in this survey were occupational therapists employed in primary care by the Region Västra Götaland and currently practicing occupational therapy (*n* = 193). This is Sweden's second largest county council, providing healthcare services to nearly 1.7 million inhabitants in western Sweden. Individuals on parental leave and long-term sick leave (*n* = 16) and those involved in development of regional clinical practice guidelines (*n* = 2) were excluded. Data were collected during November–December 2016.

### 2.2. Data Collection

Eligible participants were invited to respond to the web-based survey, using the survey software esMaker® (Entergate AB, Halmstad, Sweden). Three reminder notices were sent via e-mail during a two-month period. A statement in the survey informed the respondents that their response was assumed as informed consent. The survey was filled out anonymously, and responses could not be traced back to the respondents. According to Swedish law, ethical approval for this type of research was not required.

Questions for the survey were drawn from a validated EBP questionnaire, originally developed by Jette et al. [19] to examine EBP and guideline variables among American physiotherapists. The questionnaire had been previously translated, adapted, and validated for a Swedish primary care context [20] and used to survey Swedish physiotherapists [21]. It was for the present study minimally modified for use with occupational therapists in the same context. The questionnaire comprised eight items on participant characteristics and 23 items reflecting various aspects of EBP and guidelines in the following domains: attitudes, knowledge, behaviour, and prerequisites and barriers related to EBP resources and guidelines. Most items were rated on 5-point Likert type scales ranging from “strongly disagree” to “strongly agree,” with a “neutral” category in the middle. The item on frequency of use of guidelines ranged from “very infrequently” to “very frequently.” The items on awareness that guidelines exist and knowing how and where to find them were answered with “yes,” “to some extent,” or “no.”

### 2.3. Data Analysis

Frequencies and distributions were analyzed with descriptive statistics. Bivariate logistic regression analyses were performed to explore associations with demographic variables. Associations between attitudinal, knowledge, and behavioural variables and frequent guideline use were also explored. Variables identified as significant in univariate analyses were tested for correlation against a criteria of Spearman's rho < 0.7 to qualify for inclusion in a multivariable model. They were entered in a multivariable model, using the stepwise forward conditional method. Interaction effects were tested at the 1% level.

Before analysis, response categories for the dependent variable “use of guidelines” were dichotomised into “frequent use” versus “infrequent use” (including “sometimes”) and for independent variables into “agree” versus “disagree.” Responses for the items with 3-point scales were dichotomised into “yes” versus “no/to some extent.” The significance level was set to 0.05. Missing data were handled with listwise deletion. Statistical analyses were performed using IBM SPSS version 22.0.

## 3. Results

### 3.1. Respondents

Of the 175 occupational therapists invited to participate, 94 responded: response rate 53.7%. Participant characteristics are shown in Table 1. Internal missing values ranged from 0 to 5 (0% to 5.3%).

### 3.2. Attitudes, Knowledge, and Behaviour Related to EBP

Response frequencies for all variables are presented in Table 2. Most respondents agreed that EBP is necessary to practice (97%) and helps in decision making (92%). Approximately half of the respondents disagreed that EBP creates unreasonable demands (51%). Most (97%) agreed to wanting to improve their skills to apply EBP in their practice. Fifty-two percent agreed to feeling confident in their ability to find relevant research for their clinical questions, and 56% reported feeling confident about treating patients according to current best evidence.

Fifty percent stated that they agreed that the use of research was encouraged at their workplace, whereas 32% disagreed. Forty-six percent agreed that they knew how to access databases through the electronic library, whereas 45% disagreed.

Eighty-two percent of the respondents reported reading fewer than two articles and the rest of the respondents (19%) read 2–5 articles in an average month. Ninety percent reported performing fewer than two, 9% 2–5, and 1% 6–10 database searches per month on average.

### 3.3. Attitudes, Knowledge, and Behaviour Related to Guidelines

All of the respondents agreed that it is important to use guidelines. Sixty-five percent stated that they were aware of guidelines relevant to their work and 32% that they were partially aware. Nearly half of the respondents (47%) knew where to find guidelines on the Internet, and 44% knew this to some extent. Eighty-one percent agreed to having easy access to relevant guidelines at their workplace. Seventy-five percent reported knowing how to integrate patient preferences with guidelines.

All of the respondents agreed that guidelines are important for the patient to get the best treatment possible. Ninety-five percent agreed that guidelines facilitate their work, and 99% agreed that guidelines are important so that patients receive equal treatment. Seventy-five percent of the respondents reported using guidelines frequently or very frequently, 21% sometimes, and 3% infrequently or never.

Therapists who perceived that they had easy access to guidelines, as well as those who felt confident in their ability to treat patients according to evidence, were more likely to use guidelines frequently (Table 3). Other variables that were associated with frequent guideline use in univariate analyses did not remain significant in the final multiple regression model. No significant interaction effects between those variables and demographic variables were found.

**Figure 1:**
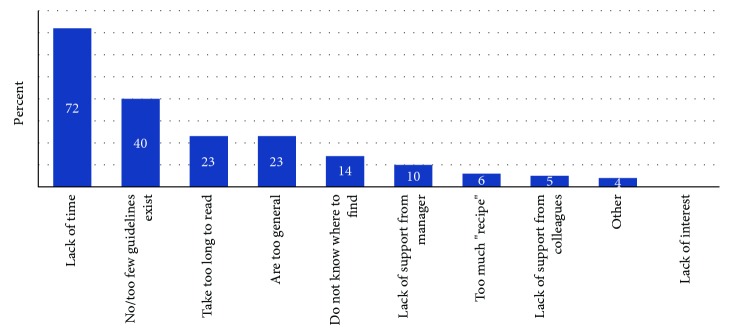
Reported barriers for using guidelines.

Reported barriers for using guidelines are shown in Figure 1. The most important barrier was lack of time, cited by 72% of the respondents. This was followed by a perceived paucity of guidelines relevant for primary care occupational therapy, that it takes too much time to read guidelines, and that guidelines are too general and unspecific. The category “too much recipe” represents the perception that many patients do not fit a specific guideline, e.g., due to comorbidity or other complex situations.

### 3.4. Associations with Demographic Variables

Few demographic characteristics were associated with EBP and guideline variables. Men were more likely than women to report self-efficacy in finding research (OR 8.58, 95% CI 1.03 to 71.66; *p* = 0.047) and having easy access to guidelines (OR 9.10, 95% CI 1.94 to 42.83; *p* = 0.005). Occupational therapists older than 50 years were more likely to report knowing how to integrate patient preferences with guideline use than younger therapists (OR 6.44, 95% CI 1.14 to 36.57; *p* = 0.035). No significant association was found between EBP variables and therapist experience.

## 4. Discussion

The study provides new knowledge on EBP and guideline use among primary care occupational therapists. Key findings of this study are that attitudes toward EBP and guidelines were highly positive and that a large majority of the respondents were aware of relevant guidelines and knew where to find them. Four-fifths reported having easy access to guidelines, and three quarters claimed to use guidelines frequently. The largest barrier for using guidelines was lack of time. Two factors were found to predict frequent guideline use: feeling able to treat patients according to evidence and having easy access to guidelines.

Although the vast majority of the occupational therapists had very positive attitudes to both EBP and guidelines, EBP-related behaviour did not reflect these attitudes. Only a small proportion said that they searched the literature and read scientific articles. These findings may be related to EBP not being taught consistently at undergraduate occupational therapy programs. The reported high awareness of guidelines, knowledge of where to find them, and easy access to them were likely related to a guideline project that had been ongoing within the county council over the past few years. That project involved the development of several practice guidelines that were published on a dedicated website, in order to make the guidelines easily accessible. Awareness and access are important prerequisites of guideline use, which in fact also was quite high; three quarters of the respondents reporting using guidelines frequently, with another fifth using guidelines sometimes.

The finding that men were more likely than women to report self-efficacy to find research and having easy access to guidelines needs to be interpreted with caution, because gender distribution of the sample was quite skewed, less than one-tenth were men. In addition, the confidence intervals for the odds ratios were quite wide. The finding that older occupational therapists were more likely to know how to integrate patient preferences with guideline use may be understandable, in that younger therapists did not have as much experience with different kinds of patients and therefore may not be skilled in incorporating their preferences into therapy.

Only two variables, self-efficacy to treat patients according to evidence and having easy access to guidelines, remained significantly associated with frequent use of guidelines in the final regression model. While easy access to guidelines is an obvious prerequisite of using them, feeling confident in one's ability to treat patients according to evidence is an interesting finding and less easily explainable. The opposite would have been more logical—feeling confident to treat according to evidence would reduce the need for guidelines. However, the link may be more easily understood when considering that those who are keen on and able to treat according to evidence are probably also more likely to want to rely on guidelines that include evidence-based recommendations.

The findings of positive attitudes toward EBP but limited perceived knowledge and skills are supported by research from other countries. McCluskey [9] reported that half of the occupational therapists in an Australian survey rated their level of EBP knowledge and skills as low. Lyons et al. [4] reported positive attitudes towards research among Australian paediatric occupational therapists, but poor confidence in research knowledge and in how to implement research findings into practice. In South Africa, Buchanan [22] showed positive perceptions toward EBP among occupational therapists but poor confidence in EBP skills, attributed primarily to limited knowledge and skills.

In our study, most respondents estimated their EBP knowledge from moderate to high; yet nearly all expressed a desire to learn more about EBP. Similar findings were shown in the study by Lyons et al. [4], where the respondents held positive attitudes toward research and wanted to access new information to guide practice.

Our study suggests a gap between positive attitudes towards EBP and sufficient knowledge to apply EBP by searching for and reading scientific literature. This gap was also identified by Upton et al. [5], who found that occupational therapists across several countries generally hold positive attitudes toward EBP but that these attitudes do not necessarily translate into practice. Salls et al. [8] showed that although a large majority of American occupational therapists considered EBP important to their practice, use of research findings to inform practice remained limited.

Our study, like most studies on EBP in occupational therapy, was conducted among practicing clinicians. In students, it is likely that search and appraisal skills are taught already in undergraduate training and that both attitudes and behaviours related to EBP therefore are more positive than those of practicing therapists. Stronge and Cahill [3] found that students in Ireland had a clear understanding of EBP and were willing to practice EBP in their future and that many accessed evidence on a weekly basis. However, Crabtree et al. [23] showed that although a master-level EBP training program improved basic EBP skills and knowledge among Canadian students, those skills were not retained after eight weeks of fieldwork. To support both students and recent graduates entering clinical practice, those findings, as well as the findings of our study, underscore the importance of continued training of clinicians in EBP skills.

Occupational therapists are expected to base their practice on the best available evidence [24, 25]. In our study, half of the respondents disagreed that applying EBP in daily practice is an unreasonable requirement, representing a positive attitude to EBP. However, nearly one-third considered it an unreasonable requirement. The reason for this negative attitude is not known, but a plausible explanation is a perceived lack of time, which was identified as a major barrier to EBP.

The multiple barriers identified for using EBP and guidelines are consistent with previous research. Lack of time was by far the most important barrier, and a quarter of the respondents also stated that it took too much time to read guidelines. Upton et al. [5] identified lack of time, availability and accessibility to research, and limited research skills as main barriers for applying EBP among occupational therapists. Lyons et al. [4] reported lack of time to read research and to implement new ideas as the greatest barriers for applying EBP in clinical practice among Australian occupational therapists. Lack of skills to understand statistics and to evaluate research quality was also among the top perceived barriers. Among South African occupational therapists, reported barriers include lack of time, knowledge, and convenient access to evidence [22]. In US occupational therapists, lack of time, high continuing education costs, weak research analysis skills, and placing higher value on clinical experience than on research have been reported as import barriers [26]. In Sweden, Heiwe et al. [13] identified lack of time, statistical knowledge, and research and appraisal skills as important barriers for EBP among hospital-based occupational therapists. Similar barriers have also consistently been reported in other healthcare professions, for example, physiotherapists [21, 27, 28], physicians [29, 30], and nurses [31].

The finding that some therapists felt that guideline use was too “recipe-like” may be indicative of the complexity of clinical reasoning and decision making and why therapists may not be using research evidence as the predominant method of achieving EBP. Planning individual therapy requires integration of research evidence with a range of different information sources such as the patient and their circumstances and experiences and the setting, in order to be both evidence-based and client-centred. Clinical reasoning, involving the thinking process of planning, conducting, and reflecting on clinical practice [32], is an essential part of making decisions about occupational therapy service delivery. It often draws on factual knowledge, tacit and practical know-how, and gut response to clients and their situations in an attempt to synthesize these different modes through metacognition [33]. Clinical reasoning is a skill that evolves with experience and has been suggested to differ between novice and experienced occupational therapists [34]. Even though our study failed to show any differences related to age or experience, it is likely that older and more experienced therapists rely more on their experience and less on research evidence than do their younger colleagues. Integrating the three EBP components is not self-evident. The research evidence component is conceptually different from the other two components; it denotes published, collective evidence, whereas clinical expertise and patient preferences mostly relate to the individual level [35]. Viewed in this light, the level of guideline use showed in this study forms a solid basis for integration with the other components. Treatment decisions should be made jointly with the patient, and their preferences and values should be elicited as part of this dialogue so that treatment can be adequately individualised and person-centred. This is particularly important in occupational therapy where several treatment options may be appropriate and research evidence for some treatments remains limited [36]. Communication and a good patient-therapist dialogue then become even more important and lay the ground for collaborative rehabilitation [37].

The history and tradition of occupational therapy is another barrier that may impede EBP. Occupational therapy is a profession that tends to rely on professional craft and personal knowledge which may reduce the importance attached to EBP [38]. Welch and Dawson [38] found that research knowledge was not included in British occupational therapists' construct of a good practitioner and that they lacked confidence in EBP. However, they also showed that practice-embedded collaborative learning was a way to develop this confidence and facilitate the therapists' use of research knowledge in their clinical reasoning. Furthermore, as suggested by Miller and Willis [11], the occupational therapy profession has produced less research in comparison to other health professionals such as physicians, nurses, and physiotherapists, which may constitute a significant barrier for occupational therapists to adopt and apply EBP. The low volume of research produced likely contributes to the low evidence grade for many occupational therapy interventions, the paucity of clinical practice guidelines, and to the fairly high proportion of respondents in our survey who did not feel confident in finding and using evidence to treat their patients.

In our study, nearly half of the respondents reported that guidelines were lacking for their specific patient population. Many also perceived that existing guidelines were too general in their recommendations. These are barriers related to guideline development that can and should be addressed by policy makers and guideline development teams, who could make further efforts to ensure that more relevant guidelines are developed and that they are specific in scope and recommendations.

### 4.1. Strengths and Limitations

Study limitations include a relatively low response rate and the lack of nonresponse analysis. With the exception of gender, we had no data on characteristics or outcome variables for those who did not respond to the survey. Men were overrepresented among respondents (10%) vs. nonrespondents (3%). Perhaps, those who responded to the survey were more positive toward EBP than those who did not respond. A further limitation is that, as in all questionnaire-based surveys, data were self-reported. Only publically employed occupational therapists were surveyed; attitudes and behaviour among private practitioners may differ.

A strength of the study is the use of a validated and reliable EBP questionnaire [20], which has previously been used among physiotherapists in Sweden [21]. Advantages of this questionnaire is that it targets practicing clinicians rather than students and that it, in addition to EBP variables, includes items on guidelines. Its forerunner, an EBP questionnaire developed by Jette et al. [19], has been used in a multitude of studies, particularly in physiotherapy [13, 39–42].

The study participants are likely to be similar in characteristics, attitudes, and behaviours to the national population of occupational therapists in primary care in Sweden; the findings can therefore probably be generalised to the rest of Sweden and possibly other countries with similar healthcare systems and practices. Still, the relatively low sample size calls for caution on an overly broad generalisation of the findings.

### 4.2. Implications for Practice and Research

The study findings have several implications for both clinicians and researchers in primary care occupational therapy. The prerequisites for applying EBP to a greater extent create high demands for the coming years, on both practicing therapists, their managers, as well as students and recent graduates who are entering the job market. Because today's occupational therapy, students learn EBP skills in their undergraduate training, efforts on improving EBP skills, and behaviour should focus on continued professional development efforts among practicing occupational therapists in primary care. Such practitioner training should be comprehensive, contextually relevant, and collaborative to help practitioners implement and integrate EBP into clinical practice and is more likely to change practice patterns if conducted in workplace settings [43]. The importance of integrating different sources of evidence into clinical reasoning should be emphasised.

The insight provided by this study into the reported attitudes towards knowledge of and barriers for EBP and guidelines can facilitate the development of strategies for increasing the use of EBP among occupational therapists. The identified barriers related to EBP could be addressed in several ways. The barriers related to the use of guidelines would be particularly relevant to address in guideline development work: making the guidelines brief, specific, and with clear links to the underpinning evidence.

The major barrier of perceived time constraints, which could be both a personal and a contextual or organisational barrier, needs to be addressed. This barrier is not likely to be reduced without organisational or managerial support, for instance, by provision of protected work time for searching and appraising literature and other EBP activities. One such activity that may be both effective and cost-effective, in that it requires minimal resources, could be the organisation of journal clubs. This is a form of EBP teaching that allows participants to keep up-to-date with current, clinically relevant, research evidence as well as to improve their research appraisal skills. Journal clubs have been shown to be acceptable to occupational therapists and other allied health professionals and to be likely to be used with enthusiasm to achieve EBP [44].

### 4.3. Future Research

To minimise bias from self-reported data, there is a need to develop objective measures for assessing implementation of EBP. For a deeper understanding of EBP behaviours, as well as barriers and facilitators for applying EBP, a qualitative approach using individual interviews or focus groups is suggested. Furthermore, research is needed to address the remaining knowledge gaps with regard to occupational therapy interventions. Many of the interventions that occupational therapists use have yet to be underpinned by substantial research evidence, due to the limited number of studies performed as well as problems with research design.

## 5. Conclusions

This study provides new knowledge about attitudes, knowledge, behaviour, prerequisites, and barriers to EBP and guidelines among Swedish primary care occupational therapists that are likely to be transferable to other countries with similar healthcare systems. In spite of very positive attitudes and a fairly high use of clinical guidelines, the large proportion of occupational therapists who wanted to learn more about EBP, the small proportion who searched for and read scientific articles, and the fairly poor self-efficacy in finding research and in treating patients according to research evidence sends a signal to managers and decision makers that measures need to be taken to address these shortcomings. The study findings highlight the importance of continual professional development and training in EBP skills, with focus on search and appraisal of literature as well as on integrating different sources of evidence into clinical reasoning. In addition, more research is needed to address knowledge gaps that remain with regard to occupational therapy interventions.

## Figures and Tables

**Table 1:** Participant characteristics (*n* = 94).

Characteristic	*n*	%
*Sex*		
Women	85	90.4
Men	9	9.6
*Age (years)*		
20–29	7	7.4
30–39	29	30.9
40–49	22	23.4
50–59	24	25.5
>60	12	12.8
*Education level/degree*		
Lower level degree	16	17.0
Bachelor's degree	74	78.7
Master's degree	4	4.3
PhD student or PhD	0	0.0
*Certified specialist*	8	8.6
*Years of experience in primary care occupational therapy*		
<3	31	33.0
3–5	11	11.7
6–10	13	13.8
11–15	12	12.8
16–20	11	11.7
>20	16	17.0

**Table 2:** Distribution of questionnaire responses.

Variable	*n*	Response frequencies^∗^				
*Attitudes to EBP*		*Strongly disagree*	*Disagree*	*Neutral*	*Agree*	*Strongly agree*
EBP is necessary to practice	94	0 (0.0%)	0 (0.0%)	3 (3.2%)	45 (47.9%)	46 (48.9%)
EBP creates unreasonable demands	94	12 (12.8%)	36 (38.3%)	19 (20.2%)	26 (27.7%)	1 (1.1%)
EBP helps decision making	94	0 (0.0%)	0 (0.0%)	8 (8.5%)	53 (56.4%)	33 (35.1%)
Want to learn/improve skills	92	0 (0.0%)	0 (0.0%)	3 (3.3%)	34 (37.0%)	55 (59.8%)
Strong evidence is lacking for most treatments	90	6 (6.7%)	27 (30.0%)	12 (5.2%)	38 (42.2%)	7 (7.8%)
Self-efficacy to find research	94	8 (8.5%)	25 (26.6%)	12 (12.8%)	39 (41.5%)	10 (10.6%)
Self-efficacy to treat patients according to evidence	93	2 (2.2%)	18 (19.4%)	21 (22.6%)	48 (51.6%)	4 (4.3%)
*Knowledge about EBP*		*Strongly disagree*	*Disagree*	*Neutral*	*Agree*	*Strongly agree*
Know how to access databases	92	25 (27.2%)	16 (17.4%)	9 (9.8%)	31 (33.7%)	11 (12.0%)
*Behaviour related to EBP*		*≤1/month*	*2–5/month*	*6–10/month*	*11–15/month*	*16+/month*
Read articles, no. of articles per month	92	75 (81.5%)	17 (18.5%)	0 (0.0%)	0 (0.0%)	0 (0.0%)
Search databases, no. of searches per month	93	84 (90.3%)	9 (8.6%)	1 (1.1%)	0 (0.0%)	0 (0.0%)
*Prerequisites for EBP*		*Strongly disagree*	*Disagree*	*Neutral*	*Agree*	*Strongly agree*
EBP encouraged at workplace	94	10 (10.6%)	20 (21.3%)	17 (18.1%)	35 (37.2%)	12 (12.9%)
*Attitudes to guidelines*		*Strongly disagree*	*Disagree*	*Neutral*	*Agree*	*Strongly agree*
Important that guidelines exist	93	0 (0.0%)	2 (2.2%)	0 (0%)	12 (12.9%)	79 (84.9%)
Important to use guidelines	91	0 (0.0%)	0 (0%)	0 (0%)	26 (28.6%)	65 (71.4%)
Guidelines are important to facilitate practice	94	0 (0.0%)	1 (1.1%)	3 (3.2%)	41 (43.6%)	49 (52.1%)
*Knowledge about guidelines*		*Strongly disagree*	*Disagree*	*Neutral*	*Agree*	*Strongly agree*
Know how to integrate pat. pref. w/ guidelines	92	0 (0.0%)	0 (0.0%)	23 (25.0%)	59 (64.1%)	10 (10.9%)
		*No*	*To some extent*	*Yes*		
Aware that guidelines exist	93	3 (3.2%)	30 (32.3%)	60 (64.5%)		
Know where to find guidelines	93	8 (8.6%)	41 (44.1%)	44 (47.3%)		
*Prerequisites for use of guidelines*		*Strongly disagree*	*Disagree*	*Neutral*	*Agree*	*Strongly agree*
Have easy access to guidelines	92	3 (3.3%)	5 (5.4%)	10 (10.9%)	54 (58.7%)	20 (21.7%)
*Behaviour related to guidelines*		*Very infrequently or never*	*Infrequently*	*Sometimes*	*Frequently*	*Very frequently or always*
Use guidelines	93	2 (2.2%)	1 (1.1%)	20 (21.5%)	51 (54.8%)	19 (20.4%)

^∗^Data are numbers (percentages).

**Table 3:** Significant associations in univariate analyses and in the final multiple logistic regression model. Dependent variable: frequent use of guidelines.

Independent variable	Level	*n*	Univariate associations		Multiple associations		
			Odds ratio (95% CI)	*p*	*B* (SE)	Odds ratio (95% CI)	*p*
Strong evidence is lacking for most interventions	Disagree	89	3.43 (1.05–11.25)	0.042			
	Agree		Reference				
Self-efficacy to treat patients according to evidence	Agree	92	3.30 (1.23–8.87)	0.018	1.93 (0.71)	6.91 (1.73–27.60)	0.006
	Disagree		Reference			Reference	
Know where to find guidelines	Yes	92	6.57 (1.43–30.25)	0.016			
	No		Reference				
Easy access to guidelines	Agree	91	11.27 (3.50–36.36)	<0.001	2.92 (0.73)	18.57 (4.40–78.26)	<0.001
	Disagree		Reference			Reference	
Know how to integrate patient preferences with guidelines	Agree	91	4.28 (1.53–11.97)	0.006			
	Disagree		Reference				

^b^Nagelkerke *R* ^2^ = 0.42, overall correctly predicted = 75.3%, *B* = unstandardised regression coefficient, SE = standard error, CI = confidence interval.

## Data Availability

The data used to support the findings of this study are available from the corresponding author upon request.
